# Foliar Application of Protein Hydrolysate-Based Biostimulant and Herbal Extracts with Antifungal Properties in Winter Wheat Cultivation as a Strategy to Enhance Cereal Yield

**DOI:** 10.3390/ijms26115089

**Published:** 2025-05-26

**Authors:** Dorota Gendaszewska, Dorota Wieczorek, Paulina Pipiak, Katarzyna Miśkiewicz, Katarzyna Zacharska, Katarzyna Ławińska

**Affiliations:** 1 Łukasiewicz Research Network, Lodz Institute of Technology, 118 Gdanska Str., 90-520 Lodz, Poland; dorota.wieczorek@lit.lukasiewicz.gov.pl (D.W.); paulina.pipiak@lit.lukasiewicz.gov.pl (P.P.); katarzyna.miskiewicz@lit.lukasiewicz.gov.pl (K.M.); katarzyna.zacharska@lit.lukasiewicz.gov.pl (K.Z.); katarzyna.lawinska@lit.lukasiewicz.gov.pl (K.Ł.); 2 Interdisciplinary Doctoral School, Lodz University of Technology, 116 Stefana Żeromskiego Street, 90-543 Lodz, Poland

**Keywords:** phytotoxicity, fungistatic activity, foliar treatment, photosynthetic pigments

## Abstract

The aim of this study was to examine the effects of foliar application of protein biostimulants in combination with extracts from field horsetail (*E. arvense* L.), common tansy (*T. vulgare* L.), or yarrow (*A. millefolium* L.) on winter wheat. Initially, the fungistatic activity and phytotoxicity of three extract concentrations (1%, 5%, 10%) were tested on reference plants. The average results indicated a decrease in root elongation stimulation with increasing concentrations of all extracts. Antimicrobial tests revealed that the 5% and 10% extracts exhibited the strongest activity, especially against *S. griseus*, whereas the 1% extracts showed no inhibitory effect. The 5% concentration was chosen as optimal due to its comparable efficacy to a reference fungicide. Subsequently, the impact of combining foliar application of extracts with protein biostimulants on wheat seedling and root length, chlorophyll fluorescence, photosynthetic pigments, and soil dehydrogenase activity was analyzed. Horsetail and yarrow extracts combined with biostimulants improved plant growth, depending on dose and combination. This was particularly evident for variants S5-B8, K5-B4, K5-B8, A-B8, for which seedling lengths were 23.6 cm (16.8%), 24.4 cm (20.8%), 23.9 cm (18.3%), and 23.6 cm (16.8%), respectively. The maximum increase in chlorophyll (a + b) content (38.30%, 35.81%, and 41.24%) occurred in plants treated with S5-B4, K5-B8, and A-B4, respectively, compared with non-treated plants. In contrast, tansy extracts reduced chlorophyll content (by up to 78%) and induced moderate stress. The research highlights the potential of natural fungicides to protect plants effectively while minimizing environmental and human health risks compared to conventional chemicals.

## 1. Introduction

In recent years, research into renewable sources of agricultural biostimulants has become increasingly important. This growing interest is driven by the need for sustainability, reducing chemical pesticides, and improving the efficiency of agricultural production while taking care of the environment. As a result, biostimulants of natural origin are being intensively researched as an alternative to traditional chemical substances used in agriculture [1]. They are commonly classified into several major groups, including seaweed extracts, microbial inoculants, humic and fulvic acids, as well as protein hydrolysates and free amino acids [2]. These compounds act through diverse physiological and molecular mechanisms. Seaweed-based products enhance nutrient uptake, yield, and stress tolerance in crops due to their content of trace elements and plant growth regulators such as auxins, gibberellins, and cytokinins. Foliar application of such agents has been shown to positively affect plant growth [3,4,5]. Another important group of natural biostimulants are microbiological inoculants, which, when applied to seeds, plant surfaces, or soil, also support plant growth. The beneficial interaction of microorganisms with higher plants occurs by providing essential nutrients and producing active substances, which contributes to an increase in root mass, the accumulation of plant biomass, and the improvement of soil structure [6]. Humic substances are also important groups of biostimulants, formed through humification—the decomposition of plant and animal residues in the soil [7]. Treatment with fulvic acid has been shown to increase the rate of photosynthesis, transpiration, and intercellular CO_2_ concentration, all of which are associated with enhanced plant growth [7]. Protein-based biostimulants, including amino acids and protein hydrolysates, are also widely used in agriculture due to their ability to enhance plant growth, improve stress tolerance, and boost crop productivity [2].

Protein hydrolysates are obtained through enzymatic, chemical, or thermal hydrolysis of organic residues [8,9]. The production of biostimulants from industrial waste eliminates the problem of managing this type of waste, which is normally stored or incinerated [10]. This approach is in line with a closed-loop economy [11], as it not only reduces waste but also provides valuable biostimulants that play a crucial role in enhancing plant growth. Protein hydrolysate-based biostimulants stimulate physiological processes in plants, enhancing growth, development, and overall condition. They mainly consist of protein hydrolysates (mixtures of polypeptides and free amino acids) or free amino acids, e.g., glutamate, glutamine, proline, and glycine [8,12]. Amino acids are absorbed through both the leaves and roots, and when used as a seed pre-treatment, they can significantly influence nitrogen metabolism in plants, thereby increasing productivity [13]. Protein hydrolysates have a positive effect on the total root surface area and length, leading to more efficient use of soil nutrients and water [14,15]. For economically significant crops, this sustainable approach not only reduces waste but also offers an effective solution to enhance plant growth and productivity.

This work is a continuation of previous studies on the effects of foliar applications of biostimulants containing collagen and keratin hydrolysates with the addition of commercial fungicide containing azoxystrobin [16]. The study showed that the new products had a beneficial impact on the growth of wheat seedlings in pot experiments, as evidenced by increases in both the plant length and fresh weight. The strongest effect was observed with the formulation that contained collagen and keratin hydrolysates and sodium salicylate. Promising results suggesting the potential of combining biostimulants with fungicides, coupled with the increasing demand for natural alternatives, prompted further research into plant extracts as a possible substitute for conventional fungicides. This trend is driven by the need for more sustainable agricultural practices that reduce reliance on chemical pesticides, minimize environmental impact, and enhance crop resilience. Numerous publications in this area clearly highlight the urgent need for progress and the importance of this topic [17,18]. Crops are constantly attacked by pathogens both before and after harvest, often causing economically significant yield losses. The main culprits of plant diseases are pathogenic fungi, which result in reduced yields and reduced nutritional and organoleptic value. The use of synthetic fungicides, which is still the most effective method of protecting cereals, results in the long-term persistence of pesticide active ingredients in food and the environment [15]. In this context, the search for new solutions to effectively control these pathogens and minimise the negative impact on crop yield and quality is crucial.

Plants have unique biosynthetic capabilities that make them valuable in the production of biological compounds and as alternatives to synthetic chemicals. Natural bioactive compounds, often referred to as natural fungicides, are non-specific with a broad spectrum of activity against pathogens [19]. These extracts can act as antimicrobial agents against several fungal phytopathogens, including those from the genera *Alternaria* sp. (*A. solani*), *Aspergillus* sp. (*A. fumigatus*, *A. niger*), *Trichoderma* sp., and *Fusarium* sp. (*F. solani*, *F. oxysporum*) [20,21,22]. They can also be used against bacterial plant pathogens such as *Pectobacterium carotovorum*, *P. atrosepticum*, and *Agrobacterium tumefaciens* [17]. In addition, natural bioactive compounds not only limit fungal growth but also enhance plant defense mechanisms [19,23]. Horsetail (*Equisetum arvense* L.) is a natural resource with significant potential for use in plant protection. Its value as a fungicide has been recognised for many years. Extract from horsetail inhibits the germination and sporulation of fungal seeds, thereby preventing the spread of fungal diseases, without killing the fungi, which is typically expected from chemical pesticides. In addition to its antifungal properties, horsetail has been shown to stimulate plant resistance and defence mechanisms [24]. Common tansy (*Tanacetum vulgare* L.) is another notable plant with fungicidal properties. Recent studies have demonstrated the effectiveness of tansy extracts against various plant pathogens, including *Fusarium* sp. [19], *Candida* sp. [25], and *Penicillium* sp. [26]. What is more, tansy has been shown to possess antimicrobial and insecticidal properties, making it a versatile natural remedy in agriculture [26]. Additionally, yarrow extract (*Achillea millefolium* L.) is a valuable resource for plant protection, particularly for vines, as well as certain fruit trees and vegetables, due to its antimicrobial and antifungal properties. Its bioactive compounds contribute to reducing pathogen pressure, making it a promising component in sustainable and organic crop management strategies [27,28]. The efficacy of natural fungicides based on plant extracts depends mainly on the concentration of phenols, terpenes, and alkaloids, although the exact mechanism of action remains poorly understood [19]. The exploration of natural plant extracts as bioactive agents offers a promising and sustainable alternative to synthetic fungicides, contributing to both environmental conservation and improved crop resilience.

Considering the numerous valuable properties of natural extracts, this study aimed to evaluate the combination of a protein hydrolysate-based biostimulant with extracts from field horsetail, common tansy, or yarrow as a potential alternative to conventional fungicides in winter wheat cultivation, with the goal of enhancing cereal yield potential. Foliar application was chosen as a key tool for effective crop management, helping to mitigate the impact of abiotic and biotic stresses while supporting plant growth and increasing yields. Research to date has mainly focused on combining biostimulants with chemical fungicides, with little attention paid to the potential synergistic use of natural substances. There is still a lack of research into the combination of biostimulants with plant-based fungicides, which opens new opportunities for innovative solutions in agriculture. This approach is a promising way to achieve sustainable agriculture by reducing dependence on chemical crop protection products.

## 2. Results and Discussion

### 2.1. Screening Tests (Selection of Optimal Concentration for Pot Experiments)

#### 2.1.1. Phytotoxicity Assessment

**Figure 1:**
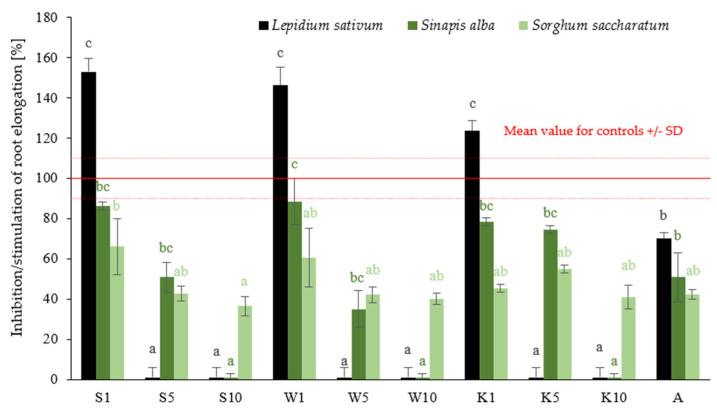
The effect of the compounds tested on the root elongation of the referenced plants: *Lepidium sativum*, *Sinapis alba*, *Sorghum saccharatum*. Values represent the mean + SD of three independent experiments, each with ten replicates (*n* = 30). For each reference plant, differences between all variants were analyzed separately with one-way ANOVA (*p* < 0.05) followed by Tukey’s post hoc multiple comparison test. Data points marked with different letters (black—*L. sativum*, dark green—*S. alba*, light green—*S. saccharatum*) are significantly different at *p* ≤ 0.05.

The results obtained in phytotoxicity tests carried out on reference plants (*Lepidium sativum*, *Sinapis alba*, *Sorghum saccharatum*) treated with extracts of the plants field horsetail extract (S), tansy extract (W), and yarrow extract (K) at different concentrations (1%, 5%, 10%) are shown in Figure 1.

The mean results show that as the concentrations of S, W, and K extracts increase, the percentage of root elongation stimulation decreases. For instance, in the case of *Sinapis alba* seeds, root growth inhibition was 86% for field horsetail extract (S) at a concentration of 1%, 51% at a concentration of 5%, and 0% at a concentration of 10%, respectively. Statistical analysis confirmed significant differences in root elongation between treatments (*p* < 0.05), with 1% extracts (S1, W1, K1) showing notably stronger stimulation. Among the studied species, *Lepidium sativum* (a dicot plant) showed the greatest sensitivity to changes in the concentration of the applied preparations—at a 1% concentration, strong stimulation of root growth was observed, while at 10%, growth practically ceased. *Sorghum saccharatum*, on the other hand, exhibited a relatively stable response, with only minor differences between concentrations. In the study by Liwarska-Bizukojc (2022), it was confirmed that cress (*Lepidium sativum*) is the most sensitive among these three reference plants, allowing for clear observation of dose-response relationships for both root and shoot length, regardless of the type of tested material [29]. It can therefore be concluded that cress is a good bioindicator for assessing the effects of xenobiotics on the early growth stages of higher plants.

The results obtained were compared with the growth inhibition values of the reference plants after the application of a 0.5% solution of the commercial fungicide Afrodyta (A). The following growth inhibition values were obtained for reference plants: *Lepidium sativum*—70%, *Sinapis alba*—51%, *Sorghum saccharatum*—42%. The analysis of these data indicates that the application of 5% solutions of plant extracts (S5, W5, K5) produces a growth effect on reference plant seedlings that is analogous to that produced by a commercial fungicide, which was statistically confirmed. The observed relationships are probably related to the chemical composition of the various extracts tested. The aboveground parts of field horsetail are a significant source of silicon (Si), which is one of the nutrients that plays a particularly important role under stress conditions, as well as improving photosynthesis and increasing nitrogen fixation [30,31,32]. Eghlima et al. (2024) confirmed that foliar application of a 2% field horsetail extract has a positive effect on the growth and oil content of basil [32]. Judžentienė et al. (2024) found that at concentrations of 0.5% and 1% of tansy extract, its effect on seed germination and the growth of garden pepper cress (*Lepidium sativum* L.) and lettuce (*Lactuca sativa* L.) was inhibitory [33]. Sousa et al. (2011) established that the aqueous extract of yarrow (*Achillea millefoilum* L.), at concentrations of 5, 10, 20, and 30 mg/mL, when treated with cells of the apical meristem of the root of lettuce (*Lactuca sativa*) in cytogenetic studies, has been shown to reduce the mitotic index (MI), seed germination, and root development of *L. sativa* [34]. The results of the studies demonstrate that elevated concentrations of aqueous plant extracts can result in phytotoxic effects, including growth inhibition and damage to plant tissues. The findings of this study suggest that, while the tested plant extracts may have beneficial effects comparable to those of commercial preparations, their application at higher concentrations may pose a risk of phytotoxicity. This highlights the importance of carefully optimizing extract dosage for potential agricultural use.

#### 2.1.2. Antifungal Properties Evaluation

The results of the antimicrobial activity of plant extracts in different concentrations and the commercial preparation Afrodyta 250 SC (A) are shown in Table 1.

**Figure 2:**
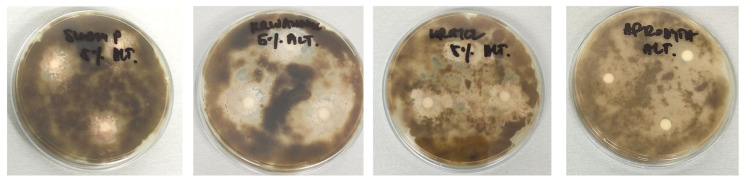
Antimicrobial activity of tested compounds against *Alternaria* spp. assessed by the disk- diffusion method. The plate shows inhibition zones for compounds S5, W5, K5, and A (from left to right).

The antimicrobial activity of plant preparations was tested against three common soil and plant pathogens. The results of the tests differed significantly depending on the type of strain used and the type and concentration of the plant preparation. The most effective were 5% and 10% extracts, particularly against *S. griseus*, while 1% extracts showed no inhibition. Against *Alternaria* sp., the 5% and 10% yarrow and tansy extracts were most effective, showing inhibition zones up to 23.33 mm. Slightly worse results were observed for field horsetail: the 5% extract inhibited *Alternaria* sp. by 16.33 mm, the 10% extract by 19.5 mm, while the 1% extract had no effect. For *F. keratoplasticum*, no antimicrobial properties were shown by any concentrations of horsetail extracts, as well as by 1% extracts of tansy and yarrow. However, the best effects were obtained for the yarrow extract at concentrations of 5% and 10%. Tansy preparations showed similar but slightly worse results. The comparison of the antimicrobial effect of the tested extracts at a concentration of 5% and the fungicide is shown in Figure 2.

A similar study on the antifungal properties of herbal extracts was conducted by Kursa et al. (2022a) [19]. They examined the effects of 5%, 10%, and 20% extracts of tansy, yarrow, and horseradish on *A. alternata*, *B. cinerea*, *C. coccodes*, and *F. oxysporum*, demonstrating that fungal inhibition varied depending on the fungus species, extract type, concentration, and exposure time. Kursa et al. (2022b) evaluated the impact of different tansy extract concentrations on the linear growth of *F. avenaceum*, *F. culmorum*, *F. graminearum*, and *F. sporotrichioides.* It was found that 20% of the tansy extract exhibited strong antifungal activity (with a maximum inhibition coefficient of 72.58%), while 10% and 5% extracts were less effective against *Fusarium* [35]. The study by Boligłowa et al. (2003) focused on aqueous plant extracts and macerates of yarrow, horsetail, chamomile, plantain, and couch grass rhizomes and their effect on the growth of *Fusarium culmorum* and *F. solani* incubated at 18 °C. The results showed that the *F. culmorum*, unlike *F. solani*, proved more sensitive to macerates than the aqueous plant extracts used [36]. It may be worth considering this method of obtaining extracts in further work. The research presented in this paper has proven that plant extracts of horsetail, tansy, and yarrow exhibit antimicrobial activity against plant pathogens, but this is dependent on the concentration of the extract and the sensitivity of the strain. Based on the results, a concentration of 5% of the extracts was selected for further work, which was found to be effective at inhibiting the growth of the plant pathogens tested.

### 2.2. Results of Pots Experiments

#### 2.2.1. Effect of Foliar Application of Tested Preparation on Winter Wheat Growth

**Figure 3:**
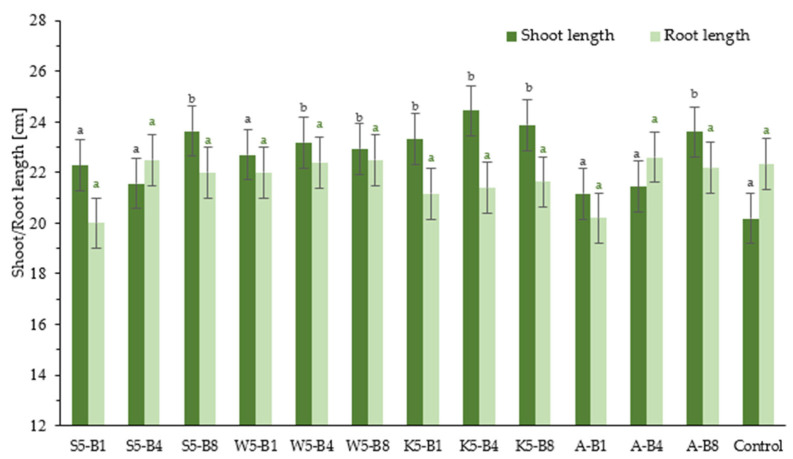
Effect of foliar application of tested substances on shoot and root length growth in winter wheat. Values represent the mean of 10 analyses per variant ±SD. Analysis of variance (ANOVA) for all parameters was followed by the Tukey post hoc test and statistically significant differences from control (*p* ≤ 0.05) are marked using low letters (black—shoot length, green—root length). Statistical analysis was performed for each parameter (shoot or root length) separately.

The impact of the tested foliar preparations on selected growth parameters, such as the length of wheat shoot and root, is illustrated in Figure 3. The grow-box experiments mainly showed that in all the variants tested, seedling length was greater compared to the control group, while root length did not differ significantly between the trials tested. Seedling lengths were 21.2–24.4 cm for the test trials and 20.2 cm for the control group (sprayed with water only). Root lengths for all trials ranged from 20 to 22.6 cm. The experiment results showed that some of the variants tested had higher shoot length values compared to others, with differences observed at a statistically significant level (*p* < 0.05). This was particularly evident for variants S5-B8, K5-B4, K5-B8, and A-B8, for which seedling lengths were 23.6 cm (16.8%), 24.4 cm (20.8%), 23.9 cm (18.3%), and 23.6 cm (16.8%), respectively. Following the introduction of the B8 biostimulant in conjunction with various natural extracts, the optimal outcomes were attained. The B8 biostimulant formula (collagen and keratin hydrolysate, sodium salicylate) demonstrated a marked effect on shoot growth. The results obtained in this study are consistent with the limited data available in the literature on the effects of keratin application on plant growth [37,38]. The study by Gaidau et al. (2021) confirmed the biostimulant properties of keratin hydrolysate in maize, showing that foliar application under laboratory and greenhouse conditions increased plant height by 8.4–19% compared to the control [37]. Similarly, greenhouse experiments conducted by Metomo et al. (2024) demonstrated that hydrolysate application positively affected morphological traits of maize, such as plant height and leaf area. The magnitude of the response depended on the concentration of the applied hydrolysate [38].

The use of plant extracts may contribute to reducing the use of synthetic fungicides, which is beneficial from both an environmental and consumer health perspective. Foliar application has been found to be an effective method of delivering biostimulants to plants, as confirmed by several reports [39,40,41]. In conclusion, the experimental variants used had an influence on plant growth, and some combinations of conditions appear particularly beneficial compared to the control.

#### 2.2.2. Effect of Foliar Application of Tested Preparation on FW and DW Contents of Winter Wheat Seedlings

To further describe the effect of the biostimulants on plant growth, the fresh and dry weights of winter wheat shoots and roots in this experiment were also determined. Significant changes in fresh weight (FW) were observed in some treated shoots compared to the control (Table 2).

There was a statistically significant increase in shoot FW in plants treated with S5-B1 (about 40% compared to the control), as well as a slight increase in K5-B8 (about 16% compared to the control) and A-B8 (about 20% compared to the control). In these cases, shoot dry weight (DW) was also slightly higher, but not at a significant level. Unexpectedly, shoot FW in plants treated with A-B1 was significantly lower compared to the control group. Furthermore, there were no significant differences in fresh and dry root weight between the tested variants and the control, suggesting that the formulations used did not have a significant effect on biomass accumulation in these plant parts in the first 31 days of cultivation. In the study, plants treated with biostimulant B8 (with a plant extract/fungicide), containing, among others, keratin hydrolysate, showed an increase in shoot FW. The observed relationship indicates that the appropriate selection of the amino acid profile of the protein biostimulants used is of particular importance in improving plant growth. Gaidau et al. (2021) and Możejko et al. (2023) confirmed the biostimulatory properties of foliar application of keratin hydrolysate in *Zea mays* (maize) [37], *Lepidium sativum* L. (garden cress), and *Brassica napus* L. var. napus (oilseed rape) plants [42]. The amino acid profile of the keratin hydrolysate revealed the presence of glutamic acid, valine, leucine, isoleucine, threonine, serine, methionine, and phenylalanine, all of which have well-documented roles in supporting plant growth and development [8,37,42].

#### 2.2.3. Variations in the Content of Photosynthetic Pigments

**Figure 4:**
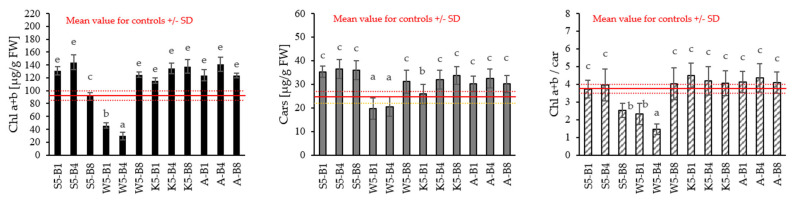
Content of chlorophyll a (Chl a), chlorophyll b (Chl b), chlorophyll a + b (Chl a + b), carotenoids (Cars) in leaf of 31—day winter wheat. Values followed by different letters are significantly different (*p* < 0.05; ANOVA followed by Tukey’s post hoc test; *n* = 10).

The analysis showed some significant changes in chlorophyll a + b (Chl a + b), carotenoids (Cars), and in the ratio of chlorophyll a + b and carotenoids (Chl a + b/car) (Figure 4).

Most variants showed relatively high chlorophyll (a + b) content compared to the control. The maximum increase in chlorophyll (a + b) content (38.30%, 35.81%, 41.24%) occurred in plants treated with S5-B4, K5-B8, and A-B4, respectively, compared with non-treated plants. The exception is plants treated with tansy extract, for which chlorophyll (a + b) levels are significantly lower, by up to 78%. Similar observations were made for carotenoids (Cars), for which higher values were recorded for plants treated with horsetail extract (44–48% higher than the control) and yarrow (30–33% higher than the control), and lower for plants treated with the tansy extract (16–19% lower than the control). The results of the Chl (a + b) to carotenoids ratio confirm these observations, where the values for the preparations of the tansy extract differ significantly from the control values. This indicates a significant effect of this extract on the pigment composition of plants, which may suggest changes in photosynthetic efficiency or protective mechanisms against environmental stress induced by this extract.

The work of Judžentienė et al. (2024) confirms these observations, highlighting the role of allelopathy in plant interactions [33]. Extracts from the aboveground parts of *T. vulgare* showed strong allelopathic effects, especially on model plants, inhibiting the germination and growth of lettuce and peppercress seeds. The strongest effect was observed on leaves, where, at concentrations of 0.5 and 1.0%, the decrease in growth was 98.46% and 89.93%, respectively. An inhibitory effect against reference plants, in this cress, was also observed in our work (see Figure 1). This is due to the presence of various allelochemicals in the extracts from the leaves of the tansy, which show strong inhibitory effects on plant growth [25]. For the other extracts, the ratio of Chl (a + b) to Cars was at a similar level as for the control.

#### 2.2.4. Variations in the Chlorophyll Fluorescence Parameters

**Figure 5:**
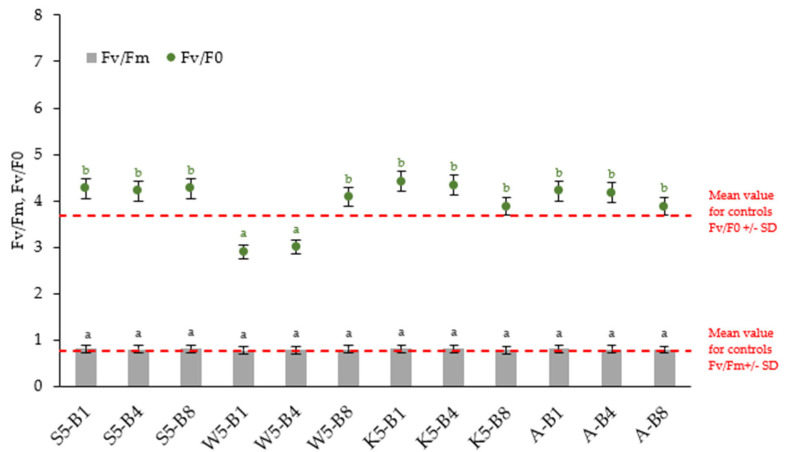
Changes in chlorophyll fluorescence parameters in 31—day winter wheat plants exposed to different preparations. Abbreviations: Fv/Fm—maximum photochemical quantum yield of PSII in the dark-adapted state, Fv/F0—efficiency of the water-splitting complex on the donor side of PSII. Within each biostimulant treatment, values with different letters (black for Fv/Fm and green for Fv/F0) are significantly different (*p* < 0.05; ANOVA followed by a Tukey’s post hoc test; *n* = 10).

Small changes in chlorophyll-a fluorescence were observed in Figure 5. The Fv/Fm ratio, indicating the maximum photochemical quantum yield of PSII, is virtually the same for all variants (0.78–0.81). Pipiak et al. (2024) and Guidi et al. (2019) noted that values between 0.75 and 0.85 are considered normal for non-stressed plants [8,43]. However, analysis of PAM chlorophyll fluorescence signals revealed significant changes in the balance between photochemical and non-photochemical processes in PSII (Fv/Fo) in some variants. The Fv/Fo ratio is generally more sensitive because it expresses the efficiency of the water-splitting complex on the donor side of PSII, which is the most sensitive component in the photosynthetic electron transport chain [44]. This parameter provides similar basic data but has higher values and a wider dynamic range than Fv/Fm [45]. This is consistent with the present results, especially for seedlings treated with tansy extract together with biostimulants (W5-B1, W5-B4), where Fv/F0 values, although like the control, remain lower than for the other variants. These results confirm previous observations indicating the occurrence of moderate stress under the cultivation conditions analyzed with tansy extract applications.

#### 2.2.5. Variations in the Dehydrogenase (DHs) Activity

**Figure 6:**
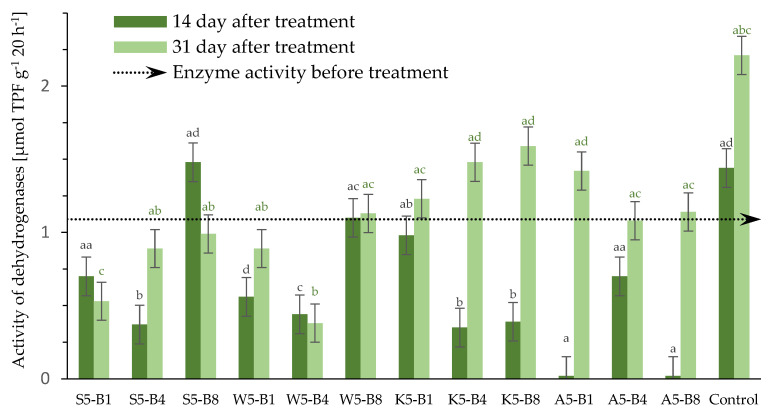
Effect of added foliar preparation on dehydrogenase activity in winter wheat cultivation collected at fixed times (1 day, 14 days, and 31 days). Values represent the mean of 3 measurements per variant at each time point ±SD. Analysis of variance (ANOVA) for all parameters was followed by the Tukey post hoc test, and statistically significant differences (*p* ≤ 0.05) are marked using low letters. Statistical analysis was performed for each time period (black letters—14, and green letters—31—day) separately.

Soil enzymes are involved in many biochemical processes. These include dehydrogenase enzymes, which catalyze the removal of hydrogen atoms from substrates during the oxidation process [46]. The role of DHs in biological oxidation processes in soil is significant. The activity of these enzymes is a reliable indicator of the presence of physiologically active microorganisms. Furthermore, DHs have been shown to be strongly associated with carbon cycles, soil organic matter (SOM), fungicide side effects, and the presence of biostimulants [47,48]. Soil dehydrogenase activity was analyzed three times during a 31-day winter wheat cultivation experiment. The experiment took place at an approximate temperature of 21/19 °C and 50% relative humidity (see point 3.2.7 for details). The control sample was soil with native microflora, to which no treatments were added. Figure 6 shows the activity of soil dehydrogenase before and after treatment with different preparations.

Soil dehydrogenase activity changes during cultivation; the highest levels were recorded for the control soil sample and soil sample treated with 5% yarrow extract, regardless of added biostimulants. These values were 2.21 μmol TPF g^−1^ 20 h^−1^ for the control sample and 1.58 μmol TPF g^−1^ 20 h^−1^ for sample K5-B8 on day 3. Enzyme inhibition was observed after 14 days. The lowest activities were in soil with the Afrodyta-based preparation at 0.02 μmol TPF g^−1^ 20 h^−1^ on day 14 in A-B1 and A-B8. However, it was observed that activity increased significantly on the 31st day of testing. This indicates a negative effect from the chemical preparation but not a long-term effect. In contrast, some natural extracts showed low activity even after 31 days. Examples of these include horsetail (*Equisetum arvense* L.) and tansy (*Tanacetum vulgare* L.) extracts. Furtak and Gajda (2017) confirmed that the presence of dehydrogenase activity indicates viable microorganisms. These have been found to correlate with organic carbon, microorganisms, nitrite, and respiration [49]. Moreover, Wang et al. (2025) established that fungicides control crop diseases caused by microorganisms but also affect soil microorganisms and enzyme activity [50]. Research studies and our own study show fungicides can reduce the number, diversity, and enzymatic activity of soil microorganisms. To reduce their toxic effects on soil, precise control of dosage is necessary. Lloyd et al. (2023) found that the effect of the fungicide can vary depending on the substance dosed at the same time [51]. This is consistent with our research. The results show the need to monitor the chemicals and biomarkers (enzymatic and otherwise).

## 3. Materials and Methods

### 3.1. Screening Tests (Selection of Optimal Concentration for Pot Experiments)

#### 3.1.1. Preparation of Herbal Extracts

Aqueous extracts were prepared from cut, dried horsetail herbs (*Equisetum arvense* L.), tansy (*Tanacetum vulgare* L.), or yarrow (*Achillea millefolium* L.) at three concentrations of 1%, 5%, and 10% (*w*/*v*), using distilled water at ambient temperature (20 °C). The selected concentrations of dissolved substances were based on literature data, where values typically range from 1% to 20% [19,23,52,53,54]. Table 3 provides an overview of the bioactive compound profiles commonly reported in the literature for aqueous extracts of horsetail, tansy, and yarrow, based on HPLC/UPLC analyses.

The herbs used in the study were obtained from a commercial supplier (Planteon, Poland). For tansy and yarrow, the entire flowering tops were used, while in the case of horsetail, only the leaves were included. Each mixture was left to stand for 48 h to complete the extraction process, after which it was filtered through filter paper. This procedure yielded nine extracts in total—one for each plant species at three concentration levels (1%, 5%, and 10%). Following filtration, the aqueous extracts were either used immediately or stored at +4 °C for up to one week [23]. All nine extracts (Table 4) were tested for phytotoxic and antimicrobial activity to determine the optimal concentration for use in grow-box experiments.

#### 3.1.2. Phytotoxicity Assessment

A phytotoxicity test based on seed germination and seedling growth of reference plants was carried out to select the appropriate concentration of plant extracts as a biofungicide in winter wheat. This was a commercial toxicity bioassay—Phytotoxkit Liquid Samples (ISO Standard 18763, Ghent, Belgium), which allows assessment of the number of germinated seeds and root and shoot growth after 72 h exposure of reference plant seeds to the samples tested [56]. These plants are the monocotyledonous plant *Sorghum saccharatum*—series no. SOS060324—and the dicotyledonous plants *Lepidium sativum*—accession no. LES270524—and *Sinapis alba*—row no. SIA171024. In this study, the concentrations of plant extracts tested were 1%, 5%, and 10% for each extract: horsetail (*Equisetum arvense* L.), tansy (*Tanacetum vulgare* L.), or yarrow (*Achillea millefolium* L.), respectively. Water was used as a control. To compare the effects of the plant extracts with those of a commercially available fungicide, a study was also carried out using the fungicide Afrodyta diluted with water (Pestila, Poland) at a concentration of 0.5%, as recommended on the product. Each sample was tested in triplicate. Ten seeds from the same test plant were placed in a single row on wet black filter paper, with equal spacing between seeds. Once assembled, the test plates were sealed, placed vertically in holders, and incubated for 72 h in the dark at 25 ± 1 °C in an incubator (DANLAB, Białystok, Poland). After incubation, digital images of each plate were taken and transferred to a computer. Root and shoot lengths were measured using ImageJ software (version 1.53k), National Institutes of Health, Bethesda, MD, USA).

#### 3.1.3. Antifungal Properties Evaluation

Natural extracts were tested for antimicrobial activity using the disk diffusion method [57]. The antimicrobial activity of the prepared biocides was tested for three fungal strains: *Streptomyces griseus* ATCC 10137, *Fusarium keratoplasticum* ATCC 36031, and *Alternaria* spp. ATCC 20084 (Pol-Aura, Poland) using the disk diffusion method. Fungal suspensions containing, respectively: 2.4–3.1 × 10^6^ cfu/mL (*F. keratoplasticum*); 3.7–4.2 × 10^6^ cfu/mL (*S. griseus*); 1.2–1.5 × 10^6^ cfu/mL (*Alternaria* spp.), 0.1 mL of a suspension of each of the three test strains was applied to the surface of a Petri dish 90 mm and spread by the stroke on Sabourand Dextrose with Chloramphenicol Lab-Agar (Biomaxima, Lublin, Poland). After absorption of the suspension, sterile blotting disks with a diameter of 6 mm soaked in the plant extracts tested (field horsetail, common tansy, and yarrow) were placed on the surface of the medium. The plates were incubated for 5–7 days at 25 ± 1 °C. After the incubation period, the growth inhibition zone around the disc was measured. At the same time, the viability of each of the three fungal suspensions was checked during the test. A sterile 6 mm tissue disc was soaked in each of the fungal suspensions, then applied to a new media plate and incubated as above. The test was performed in triplicate (*n* = 3). The positive control sample was a biostimulant preparation containing a commercially available fungicide, Afrodyta 250 SC, at a concentration of 0.5% (Pestila, Studzianki, Poland). Antimicrobial activity tests were carried out according to an in-house procedure.

### 3.2. Pot Experiments—Growth Studies on Winter Wheat

#### 3.2.1. Plant Material

The winter wheat seeds used in all studies were of the Euforia variety, obtained from the Plant Breeding Strzelce Ltd. (IHAR Group in Borowo, Main Branch in Strzelce, Strzelce, Poland). Euforia is a high-yielding winter wheat variety with excellent cold resistance. It demonstrates strong resistance to fungal diseases, particularly stem base diseases, as well as lodging and pre-harvest sprouting. Euforia wheat is characterized by a heading period of 151 days and full maturity at 204 days, showcasing a medium-early growth cycle. The sowing density varies depending on the timing, ranging from 225–300 seeds/m^2^ for early sowing (before September 20) to 350–400 seeds/m^2^ for late sowing (up to December).

#### 3.2.2. Preparations Tested

Three biostimulants, i.e., B1, B4, and B8, developed by the Łukasiewicz Research Network—Lodz Institute of Technology (Ł-LIT), were previously tested in combination with the commercially available fungicide Afrodyta 250 SC (Pestila, Studzianki, Poland) [16], demonstrating promising synergistic effects in plant protection. In collaboration with INCDTP (Romania), protein hydrolysates were produced from bovine hides with parameters typical of a semi-finished leather product (moisture content 51%, an ash content of 8.6%, a nitrogen content of 16.5%, and a pH value of 4.2). The keratin hydrolysate (78% protein, 13.9% nitrogen, pH~12) was obtained from a protein rich in sulfur, supporting plant growth. The pH of both hydrolysates was adjusted to approx. 7. Sodium salicylate (Pol-Aura, Poland) and titanium ascorbate (Łukasiewicz-LIT, Poland) were added to the preparation as biostimulants. In the present study, the same biostimulant formulations, composed of collagen and keratin hydrolysates along with other bioactive additives, were evaluated in combination with a natural extract of field horsetail (*Equisetum arvense* L.), common tansy (*Tanacetum vulgare* L.), or yarrow (*Achillea millefolium* L.) (Planteon, Poland). The extracts were selected for their documented antimicrobial properties, making them a potential alternative to conventional synthetic fungicides such as Afrodyta 250 SC. As a result, 12 different formulations with potentially dual effects—antifungal and biostimulatory—were developed. The physicochemical properties of the tested preparations were analyzed to ensure consistency and reproducibility of the formulations. The pH of each preparation was determined according to the CIPAC MT 75.3 procedure, while the density was measured using the gravimetric method, yielding values close to 1.00 g/cm^3^. Turbidity was determined using the standard method on the turbidimeter HI93414-02 (HANNA Instruments Sp. z o.o., Olsztyn, Poland). For comparison and to establish a baseline for treatment efficacy, water was used as a control for all foliar applications. Table 5 presents the characteristics of the tested formulations, including their composition and physicochemical parameters.

#### 3.2.3. Experimental Setup

The study was conducted in the grow-boxes RoyalRoom (200 × 200 × 100 cm) at the Łukasiewicz—LIT in Poland (latitude 51.79286° N, longitude 19.44788° E). The experiment was carried out in 2024. The experimental protocol was based on the comparison between twelve different preparations (Table 5). Each 10-liter pot was filled with universal agricultural soil and contained approximately 180 wheat seeds. The seedlings were sprayed with preparations on day 7 (BBCH 10) and day 14 (BBCH 12–14) with the solutions until run-off, using a hand-held sprayer (Quasar Sprayer Twister 0.5L, QUICKCLICK, Poland). The second spray was applied at the 3–4 true leaf stage. Both the lower and upper leaf surfaces were sprayed until wet, as uptake by the lower surface has been reported to be more effective. The control pot was sprayed with water only. Each treatment had three replications. The conditions for growing potted plants were described in a previous study [16]. The chemical and physical characteristics of the substrate are provided in Table 3. All seedlings were harvested 31 days after sowing. The effect of the applied preparations was studied by determining changes in growth parameters (shoot length, root length, dry and fresh weight), chlorophyll fluorescence parameters, photosynthetic pigments, and dehydrogenase activity. The chemical and physical properties of the substrate are shown in Table 6.

#### 3.2.4. Growth Measurements

The analysis was conducted two weeks after the extracts were applied to the plants. Thirty seedlings (10 plants per pot, *n* = 30) were harvested from each experimental group. The seedlings were cleaned of soil residues and weighed. Fresh weight was recorded in grams (g fw^−1^). After drying the plants in an oven at 104 °C for 4 h, the material was weighed again, and the dry weight was also recorded in grams (g dw^−1^). Additionally, the length of each seedling and root was measured, and the average seedling and root length (cm) for each applied preparation was calculated.

#### 3.2.5. Photosynthetic Pigment Content Determination

The concentrations of chlorophylls and carotenoids were determined spectrophotometrically using the method described by Porra et al. (1989) [58] and Lichtenthaler (1987) [59]. For this analysis, 1 g of plant tissue was homogenized in 10 cm^3^ of 80% acetone (Pol-Aura, Poland). After centrifugation (20 min, 20,000× *g*, 4 °C), the absorbance of the resulting extracts was measured at wavelengths of λ = 645 nm for chlorophyll a (Chl a), λ = 663 nm for chlorophyll b (Chl b), and λ = 470 nm for carotenoids (Cars). The concentrations of chlorophyll a, b, a + b, and carotenoids were calculated using Formulas (1)–(4), provided below, and expressed in μg/g of fresh weight.

Chl a = 12.25 (A663) − 2.79 (A645)(1)

Chl b = 21.5 (A645) − 5.1 (A663)(2)

Chl a + b = 18.7 (A645) + 7.15 (A663)(3)

Cars = (1000 (A470) − 1.82 (Chl a) − 85.02 (Chl b)/198)(4)

#### 3.2.6. Chlorophyll Fluorescence Analysis

Photosynthetic activity was assessed in 31-day-old winter wheat plants grown under the conditions described in Section 2.2.3. To evaluate the activity of the light phase of photosynthesis, a portable fluorimeter FluorPen FP 110/S (Photon Systems Instruments, Drásov, Czech Republic) was used to measure active chlorophyll fluorescence, followed by analysis using the NPQ test. This protocol is used for the quantitative determination of photochemical and non-photochemical quenching. The test is performed on dark-adapted samples [60]. The following parameters were determined: Fv/Fm (maximum photochemical quantum yield of PSII in the dark-adapted state) and Fv/Fo (efficiency of the water-splitting complex on the donor side of PSII). Chlorophyll fluorescence parameters were recorded at three locations on three plants from each variant. Measurements were conducted at room temperature (25 °C). The results were saved on the device and then transferred to a computer using FluorPen 1.1 software (Photon Systems Instruments, Czech Republic). This software enables data analysis.

#### 3.2.7. Dehydrogenase (DHs) Activity Test

The Casida method [61] was modified to use TTC (2.3.5-triphenyltetrazolium chloride), and a 20 h reaction [62] was used to measure soil dehydrogenase activity. Specific dyes indicate electron flow and electron transport system activity. Three grams of each soil subsample (in triplicates) were mixed with 30 mg of CaCO_3_. Next, 3% TTC (1 cm^3^) and distilled water (1.5 cm^3^) were added. The samples were then shaken and incubated at 30 °C for 20 h. 25 cm^3^ of ethyl alcohol was used for TPF (triphenyl formazan) extraction. TPF can be quantified at 485 nm. Enzyme activity was expressed in grams of µmol triphenyl formazan per g of soil within 20 h.

### 3.3. Statistical Analysis

Obtained data are presented as average values of at least three replicates in each experimental treatment. All data were analyzed by one-way ANOVA followed by Tukey’s post hoc test. Differences were considered significant at *p* < 0.05. All statistical analyses were performed using Statistica 14.1.0.4 software (TIBCO Software, Palo Alto, CA, USA).

## 4. Conclusions

The study evaluated the effect of a protein hydrolysate-based biostimulant in combination with plant extracts (from field horsetail, tansy, and yarrow) on winter wheat growth as an alternative to conventional fungicides. Plant extracts at a concentration of 5%, especially from yarrow, improved the parameters tested compared to the control, indicating their potential to reduce the use of chemicals. Particularly positive effects on shoot growth and fresh weight were observed in the treatments containing the B8 formulation, which included keratin hydrolysate. This confirms that plant biostimulation requires the presence of an appropriate amino acid profile in the biostimulant to effectively stimulate plant physiological processes. Foliar application of the biostimulant lengthened seedlings by up to 20%, while root length remained unchanged significantly. Analysis of soil dehydrogenase activity showed that plant extracts can affect soil microorganisms, with tansy and horsetail showing a long-term inhibitory effect. The results suggest that combinations of biostimulants with natural extracts, including the K5-B8 formulation, can promote cereal growth and reduce the negative effects of fungicides, although effectiveness is dose-dependent. The use of such formulations can cause both beneficial and undesirable changes, and natural extracts have not always proved to be the optimal solution. Further research, including more detailed physiological and biochemical analyses, is needed for a more complete understanding of the processes in plants treated with plant protection products. These results may have practical applications in agriculture and organic farming, especially in the context of natural weed control and sustainable crop management.

## Figures and Tables

**Table 1:** Antimicrobial activity (diameter of the inhibition zone calculated with the blotting disc in mm); mean values *n* = 3 of the tested substances at disc, TGI: total growth inhibition.

Microorganism	Extracts/Fungicide									
	S1	S5	S10	W1	W5	W10	K1	K5	K10	A
*S. griseus*	<6	TGI	TGI	<6	TGI	TGI	<6	TGI	TGI	<6
*Alternaria* spp.	<6	16.33	19.5	<6	20	23	<6	19	23.33	15
*F. keratoplasticum*	<6	<6	<6	<6	9.58	13.66	<6	13.45	17.5	10

**Table 2:** Effect of foliar application of tested preparation on growth parameters of winter wheat (FW—fresh weight, DW dry weight, control-non-treated plants). Values followed by different letters for shoot/root DW/FW length are significantly different (*p* < 0.05; ANOVA followed by Tukey’s post hoc test; *n* = 10).

Experimental Treatment	Shoot FW [g]	Shoot DW [g]	Root FW [g]	Root DW [g]
S5-B1	3.5 ± 0.5 b	0.4 ± 0.2 a	0.9 ± 0.2 a	0.3 ± 0.1 a
S5-B4	2.4 ± 0.4 ab	0.2 ± 0.0 a	0.5 ± 0.1 a	0.2 ± 0.1 a
S5-B8	2.7 ± 0.6 ab	0.3 ± 0.1 a	0.9 ± 0.2 a	0.3 ± 0.1 a
W5-B1	2.6 ± 0.6 ab	0.3 ± 0.1 a	0.9 ± 0.1 a	0.3 ± 0.1 a
W5-B4	2.5 ± 0.5 ab	0.3 ± 0.1 a	0.9 ± 0.2 a	0.3 ± 0.1 a
W5-B8	2.6 ± 0.7 ab	0.3 ± 0.1 a	0.5 ± 0.1 a	0.3 ± 0.1 a
K5-B1	2.3 ± 0.3 ab	0.2 ± 0.1 a	0.9 ± 0.2 a	0.3 ± 0.1 a
K5-B4	2.7 ± 0.3 ab	0.3 ± 0.1 a	0.9 ± 0.2 a	0.4 ± 0.1 a
K5-B8	2.9 ± 0.4 ab	0.4 ± 0.1 a	0.7 ± 0.2 a	0.3 ± 0.1 a
A-B1	1.9 ± 0.3 a	0.2 ± 0.0 a	0.8 ± 0.2 a	0.4 ± 0.1 a
A-B4	2.6 ± 0.5 ab	0.3 ± 0.2 a	0.9 ± 0.2 a	0.3 ± 0.1 a
A-B8	3.0 ± 0.4 ab	0.3 ± 0.2 a	0.6 ± 0.1 a	0.2 ± 0.0 a
Control	2.5 ± 0.2 ab	0.3 ± 0.1 a	0.8 ± 0.2 a	0.3 ± 0.0 a

**Table 3:** Composition of bioactive compounds in water extracts from *Equisetum arvense* L. (field horsetail), *Tanacetum vulgare* L. (tansy), and *Achillea millefolium* L. (yarrow).

Plant Name	Experiment Condition	Methods	Composition	Ref.
Horsetail (*Equisetum arvense* L.)	Dried leaves were ground into a powder and extracted with water containing 200 ppm SO_2_ at a 3:1 solvent-to-material ratio.	UPLC–ESI–MS–MS	Caffeoyl tartrate isomer, Caffeoylshikimic acid isomer, Dicaffeoyl tartaric acid isomer, Kaempherol diglycoside, Kaempferol-3-O-6-acetylglucoside, Quercetin dihexoside, Quercetin-glucoside, Quercetin-3-O-6-acetylglucoside isomer	[55]
Tansy (*Tanacetum vulgare* L.)	Fifteen grams of crushed herbal material with 200 mL distilled water was macerated in an ultrasonic bath at 21–24 °C for 45 min. The mixture was filtered and divided into three 25 mL portions.	HPLC-DAD-TOF	Succinic acid, Quinic acid, 3-Dehydrocaffeoyl-5-caffeoylquinic acid, 4-Dehydrocaffeoyl-5-caffeoylquinic acid, Feruloylquinic acid, Diferulic acid, Protocatechuic/gentisic acid, Ferulic (hydroxycinnamic) acid, Isochlorogenic (3,5-dicaffeoylquinic) acid A, Isochlorogenic (3,4-dicaffeoylquinic) acid B, p-Hydroxyphenylacetic acid 1-O-hexoside, Luteolin, Kaempferol, Quercetin, Acacetin, Ludovicin C, Hydroxyarbusculin, 6-Methoxykaempferol/Isorhamnetin, Isorhamnetin 3-O-glucoside, Tanacetin/armefolin, 5,7,3′-Trihydroxy-3,6,4′,5′-tetramethoxyflavone	[33]
Yarrow (*Achillea millefolium* L.)	Lyophilized plant material (1 g) was added to 200 mL distilled water, then heated and boiled for 5 min. The mixture was left to stand for 5 min, then filtered under reduced pressure. Infusions and decoctions were then frozen and lyophilized for further analysis.	HPLC	3-O-Caffeoylquinic acid, Caffeic acid hexoside, 4-O-Caffeoylquinic acid, 5-O-Caffeoylquinic acid, Apigenin C-hexoside-C-hexoside, Apigenin C-hexoside-C-pentoside, Apigenin C-glucose-C-pentoside, Luteolin 6-C-glucoside, Quercetin O-pentosyl-hexoside, Quercetin O-hexoside, Quercetin O-malonylhexosyl-rhamnoside, Kaempferol O-pentosyl-hexoside, Quercetin 3-O-rutinoside, Apigenin O-dihexoside, Isorhamnetin O-hexoside, 3,4-O-dicaffeoylquinic acid, Quercetin O-acetylhexoside, cis 3,5-O-dicaffeoylquinic acid, trans 3,5-O-dicaffeoylquinic acid, 4,5-O-dicaffeoylquinic acid, Apigenin 7-O-glucoside, Luteolin O-acetylhexoside, Isorhamnetin O-acetylhexoside, Apigenin O-acetylhexoside	[28]

**Table 4:** Tested samples in screening tests.

Code	Name	Concentration [%]	pH [-]	Density [g/cm^3^]	Turbidity [NTU]
S1	Horsetail extract (*Equisetum arvense* L.)	1	5.26	0.992	16
S5	Horsetail extract (*Equisetum arvense* L.)	5	4.40	0.995	841
S10	Horsetail extract (*Equisetum arvense* L.)	10	4.32	0.998	>1000
W1	Tansy extract (*Tanacetum vulgare* L.)	1	6.37	0.991	20
W5	Tansy extract (*Tanacetum vulgare* L.)	5	4.94	0.996	464
W10	Tansy extract (*Tanacetum vulgare* L.)	10	4.23	0.997	>1000
K1	Yarrow extract (*Achillea millefolium* L.)	1	5.80	0.990	56.1
K5	Yarrow extract (*Achillea millefolium* L.)	5	4.08	0.992	377
K10	Yarrow extract (*Achillea millefolium* L.)	10	3.99	0.998	>1000
A	Afrodyta 250 SC	0.5	7.21	0.992	>1000

**Table 5:** Physicochemical characterization of the studied dual-action formulations.

No.	Code	Name of Fungicide	Composition of Biostimulant	pH [-]	Density [g/cm^3^]	Turbidity [NTU]
1	S5-B1	Horsetail extract 5% *(Equisetum arvense* L.)	Biostimulant 1: Collagen hydrolysate 0.5% Sodium salicylate 0.03%	4.33	1.01	181
2	S5-B4	Horsetail extract 5% (*Equisetum arvense* L.)	Biostimulant 4a: Collagen hydrolysate 0.5% Titanium ascorbate 0.01%	4.32	1.01	212
3	S5-B8	Horsetail extract 5% (*Equisetum arvense* L.)	Biostimulant 8: Collagen hydrolysate 0.5% Keratin hydrolysate 0.5% Sodium salicylate 0.03%	4.32	1.02	276
4	W5-B1	Tansy extract 5% (*Tanacetum vulgare* L.)	Biostimulant 1: Collagen hydrolysate 0.5% Sodium salicylate 0.03%	5.00	1.02	30.5
5	W5-B4	Tansy extract 5% (*Tanacetum vulgare* L.)	Biostimulant 4a: Collagen hydrolysate 0.5% Titanium ascorbate 0.01%	4.99	1.02	37.0
6	W5-B8	Tansy extract 5% (*Tanacetum vulgare* L.)	Biostimulant 8: Collagen hydrolysate 0.5% Keratin hydrolysate 0.5% Sodium salicylate 0.03%	5.02	1.03	58.0
7	K5-B1	Yarrow extract 5% (*Achillea millefolium* L.)	Biostimulant 1: Collagen hydrolysate 0.5% Sodium salicylate 0.03%	4.04	1.02	471
8	K5-B4	Yarrow extract 5% (*Achillea millefolium* L.)	Biostimulant 4a: Collagen hydrolysate 0.5% Titanium ascorbate 0.01%	3.97	1.02	192
9	K5-B8	Yarrow extract 5% (*Achillea millefolium* L.)	Biostimulant 8: Collagen hydrolysate 0.5% Keratin hydrolysate 0.5% Sodium salicylate 0.03%	4.03	1.03	181
10	A-B1	Afrodyta 250 SC 0.5%	Biostimulant 1: Collagen hydrolysate 0.5% Sodium salicylate 0.03%	7.26	0.998	>1000
11	A-B4	Afrodyta 250 SC 0.5%	Biostimulant 4a: Collagen hydrolysate 0.5% Titanium ascorbate 0.01%	7.40	0.999	>1000
12	A-B8	Afrodyta 250 SC 0.5%	Biostimulant 8: Collagen hydrolysate 0.5% Keratin hydrolysate 0.5% Sodium salicylate 0.03%	7.25	0.999	>1000
13	Control	-	Water	7.00	0.998	0

**Table 6:** Physical and chemical characteristics of the soil (Nt—total nitrogen. OM—organic matter).

Salinity [g NaCl/dm^3^]	pH in H_2_O [-]	N-NO_3_ [mg/dm^3^]	P [mg/dm^3^]	K [mg/dm^3^]	Cu [mg/dm^3^]	Fe [mg/dm^3^]	Zn [mg/dm^3^]	Mg [mg/dm^3^]	C [%]	Nt [%]	OM [%]
<3.0	6.5	5.59	122	736	0.71	1.29	0.93	207	13.9	0.91	23.8

## Data Availability

The raw data supporting the conclusions of this article will be made available by the authors on request.
